# Challenges and strategy in treatment with exosomes for cell-free-based tissue engineering in dentistry

**DOI:** 10.2144/fsoa-2021-0050

**Published:** 2021-10-28

**Authors:** Ika Dewi Ana, Anggraini Barlian, Atik Choirul Hidajah, Christofora Hanny Wijaya, Hari Basuki Notobroto, Triati Dewi Kencana Wungu

**Affiliations:** 1 Department of Dental Biomedical Sciences, Faculty of Dentistry, Universitas Gadjah Mada, Yogyakarta, 55281, Indonesia; 2 School of Life Sciences & Technology, Institut Teknologi Bandung, Bandung, 40132, Indonesia; 3 Department of Epidemiology, Biostatistics, Population Studies, & Health Promotion, Faculty of Public Health, Universitas Airlangga, Surabaya, 60115, Indonesia; 4 Department of Food Science & Technology, Faculty of Agricultural Engineering & Technology, IPB University, Bogor, 16002, Indonesia; 5 Department of Physics, Faculty of Mathematics & Natural Sciences, Institut Teknologi Bandung, Bandung, 40132, Indonesia

**Keywords:** cell-free-based therapy, ceramics-based scaffold, exosome, MSC, regenerative dentistry, tissue engineering

## Abstract

In dentistry, problems of craniofacial, osteochondral, periodontal tissue, nerve, pulp or endodontics injuries, and osteoarthritis need regenerative therapy. The use of stem cells in dental tissue engineering pays a lot of increased attention, but there are challenges for its clinical applications. Therefore, cell-free-based tissue engineering using exosomes isolated from stem cells is regarded an alternative approach in regenerative dentistry. However, practical use of exosome is restricted by limited secretion capability of cells. For future regenerative treatment with exosomes, efficient strategies for large-scale clinical applications are being studied, including the use of ceramics-based scaffold to enhance exosome production and secretion which can resolve limited exosome secretory from the cells when compared with the existing methods available. Indeed, more research needs to be done on these strategies going forward.

## Lay abstract

Application of stem cells in dental tissue engineering such as in osteochondral, periodontal, pulp, salivary gland or nerve regeneration as well as in osteoarthritis, mucosal, skin and oral wound healing, is still problematic, especially for its large clinical scales. Therefore, cell-free-based tissue engineering using exosomes isolated from stem cells is regarded next-generation treatment in dentistry. In this study, basic understanding on the exosomes, status, the potential for regenerative therapy including challenges and strategies for the clinical applications are reviewed. Future approaches to increase production and secretion from the cells are also proposed to resolve limitation in exosomes availability.

## Graphical abstract

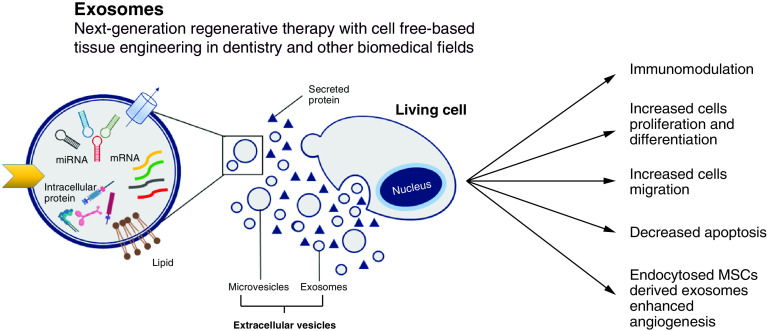

There is a limitation of the regenerative potential in human tissue. Therefore, regenerative medicine exists to stimulate and induce healing and regeneration of human tissue or organ. In regenerative medicine, tissue engineering is considered an extremely important area involving the use of materials, cells and to a large extent, signaling molecules. Tissue engineering approach has the goal to understand tissue function and enable tissue or organ on the body to be made *de novo*. To achieve very important long-range objective of tissue engineering, research in many areas with collective interdisciplinary views are required.

Based on tissue engineering paradigm, materials are necessary to design then provide proper scaffold to support the constructive remodeling of injured, damaged or missing tissues or organ. Scaffold can be engineered to have specific structural, mechanical, physical and chemical properties that closely approximate those of extracellular matrix of the tissue replaced. The scaffold should facilitate the attachment, migration, proliferation, differentiation and three-dimensional spatial organization of the cell population required for structural and functional replacement of the targeted tissue or organ [1–3]. Any scaffold materials will be subjected to *in vivo* remodeling which covers the process of host response to the scaffold materials, the degradation and replacement of scaffold by new host tissue, and the organization and differentiation of the new host tissue in relationship to surrounding structures to fully incorporate into the host toward tissue regeneration (Figure 1). The *in vivo* remodeling is influenced by several factors such as blood supply, pH, concentration of oxygen and carbon dioxide, mechanical stressors and host–scaffold interface [4,5].

**Figure 1.**
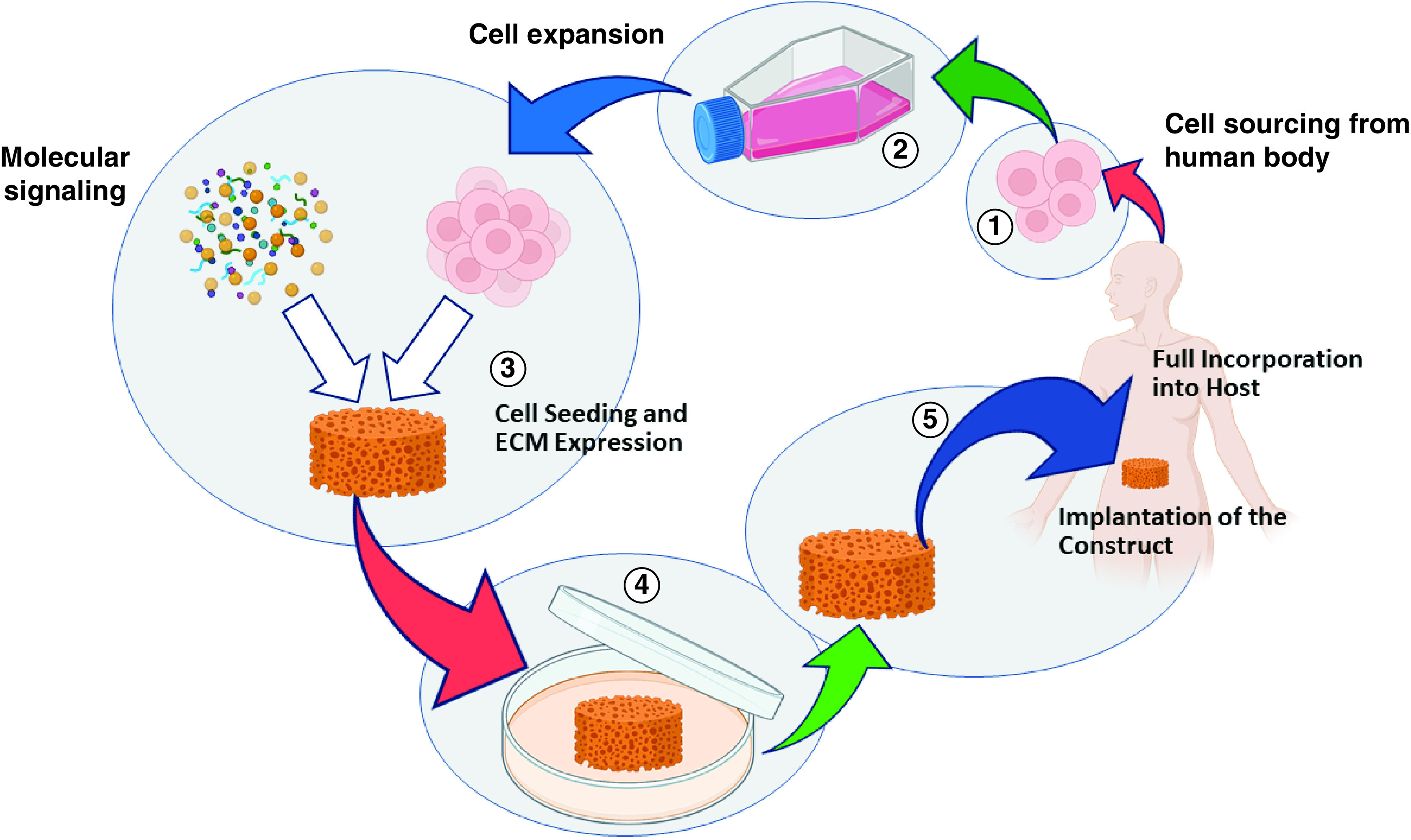
Tissue engineering paradigm with the scaffold as the central construct to provide 3D spatial for cells to attach, growth as well as organize structural and functional replacement of the tissue or organ.

The use of cells in tissue engineering attracted scientists to pay a lot of attention to stem cells, especially mesenchymal stem cells (MSCs), because their defining properties make them an ideal candidate to cure diseases. In fact, embryonic and fetal stem cells have the greatest potential to differentiate into different cell types, but their application is limited due to ethical issues and the danger of unlimited and uncontrolled cells division. The use of stem or progenitor cells have been expanded widely for treating many types of diseases in the framework of tissue engineering. It was known from previous studies that MSCs can stimulate regeneration of several tissues or organs after injury both preclinically and in clinical trials [6–15]. Mesenchymal stem cell (MSC) is a cell that has not been differentiated and able to regenerate itself through cell division, with the ability to differentiate into other cells [16]. These cells are widely used in the field of tissue engineering to regenerate bone tissue because they can differentiate into osteogenic cells [17,18].

Despite the observed beneficial effects, there is no consistent evidence that the cells employed generate organ-specific cell population, able to replace the cell loss after injury [6]. A major MSC limit for its clinical applications is the inherent heterogenicity and variation associated with cell expansion [19,20]. Changes that may increase the risk of MSC therapeutic application could also be induced during *in vitro* cell processing and expansion. The risk of unwanted differentiation *in vivo* is also a problem due to its clinical applications. Therefore, nowadays cell-free-based therapy is considered an alternative treatment in tissue engineering, which also includes extensive research on the identification of the molecules involved in paracrine action of stem cells to open new therapeutics options.

Due to the theory of paracrine stimulation, upon the application, transplanted cells will affect residing cells by secretion of bioactive molecules into the extracellular space [20]. A lot of studies have been conducted to purify growth factors (GF) and apply the GF to stimulate regeneration, including the use of platelet-rich plasma and platelet-rich fibrin [21,22]. However, the role of purified GFs in stimulating regeneration is not as effective as expected due to their short half-life in the extracellular space [23]. Therefore, there is a need to develop a strategy to overcome the disadvantages of a cell-based approach in tissue engineering and regenerative medicine. The MSCs extracellular vesicles (EVs) which contain biologically active molecules, such as GFs, cytokines and functional RNAs known as exosomes have become a particular interest for cell-free regenerative therapy due to their epigenetic capacity and cargos.

To some extent, in tissue-engineering-based regenerative therapy, there is a ‘construct’ to promote the repair and/or regeneration of tissues. As explained previously, the ‘construct’ which is provided as a scaffold and cells/biomolecules delivery system, plays an important role as a conductive strategy to interfere regenerative process by enabling the desired host cells to populate the regeneration site [3,24]. This is because scaffold is intended to support cell migration, growth, differentiation and guide tissue development and organization into a mature and healthy state [24]. Meanwhile, it is also recognized that ceramics containing construct functions as instructive extracellular microenvironment for morphogenesis [3].

In view of the current advancement and challenges, in this study, a comprehensive, hence, concise review on the role of MSCs derived exosomes in regenerative therapy applied in dentistry is elaborated. To understand the role of exosomes in regenerative therapy, overview on the origin, functions and potentials of exosomes are described. The important aspect on the osteoconductive strategy by scaffold containing ceramics is also discussed in this study.

## What is MSC-derived exosomes & why?

**Figure 2.**
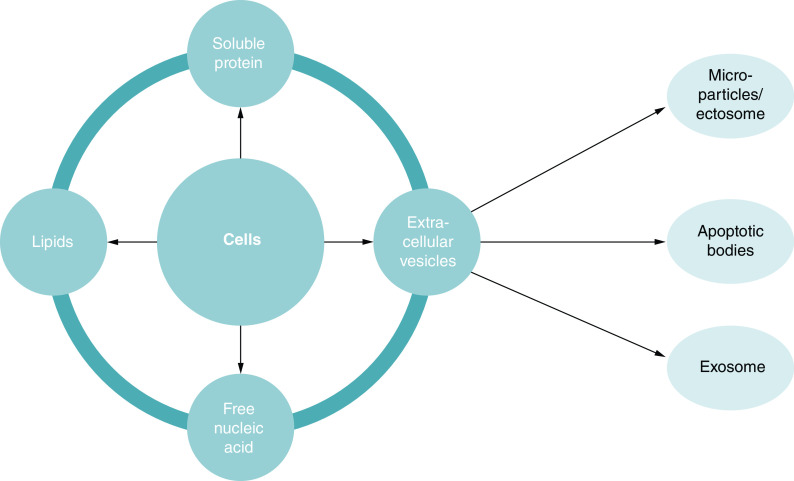
Cells secreted bodies into the extracellular space are called secretome, which contains soluble proteins, free nucleic acid, lipids and extracellular vesicles. The EVs can be divided into apoptotic bodies, microparticles and exosomes. Microparticles are also indicated with other names: nanoparticles, microvesicles, shedding vesicles or shedding bodies, exovesicles, secretory vesicles and oncosomes. Exosomes contains growth factors, cytokines and functional RNA. EV: Extracellular vesicle.

**Figure 3.**
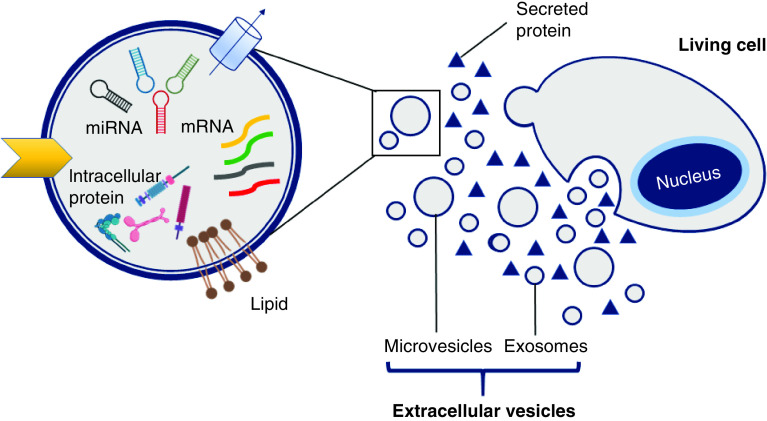
A living cell releases proteins and extracellular vesicles (microvesicles and exosomes) into extracellular spatial.

The cells (including MSC) produce a set of factors or molecules secreted to the extracellular space. The secreted factors include, among others, soluble proteins, free nucleic acids, lipids and EVs. The latter, EVs, can be subdivided into apoptotic bodies, microparticles or microvesicles and exosomes [25], which are differentiated based upon their biogenesis, release pathways, size, content and function. Table 1 shows type, size and origin of EVs [26]. Figure 2 describes diagrammatically the organization of cell secretome. Meanwhile, Figure 3 summarizes the position of exosome as a part of EVs and clarify the organization of EVs in more detailed explanation based on some of the literature and guidelines from the International Society for Extracellular Vesicles in the Minimal Information for Studies of Extracellular Vesicles 2018 (MISEV2018) on nomenclature, isolation and characterization [26,27]. The secretome of individual cells and tissues is specific, and changes in response to fluctuations in physiological states or pathological conditions. The exosome is part of the EVs originating from the endosome with a size of 30–100 nm [28]. Exosomes are known to be an important substance to facilitate cellular communication by delivering functional cargos, for example, protein, mRNA and microRNA (miRNA), as described by Wang and co-workers [29]. Exosomes from stem cells are composed of a lipid bilayer, with the ability to efficiently protect, transport, and deliver a wide variety of molecules contained in it [30]. Exosomes also play an irreplaceable role in normal physiological processes such as nerve function and neurodegenerative diseases.

**Table 1.** Types, size and origin of extracellular vesicles according to the MISEV 2018.

Types	Size (nm)	Origin
Exosome	50–100	Endosome
Micro vesicles	100–1000	Plasma membrane
Apoptotic bodies	1000–5000	Plasma membrane

Data taken from [26].

The exosomes isolated from MSCs have a more effective therapeutic ability due to their small size and role in cell communication. According to Chopra and co-workers [31], the effectiveness of exosomes derived from MSCs may be due to its ability to migrate to the areas which need repair so that they are ideal for therapeutic use. At this point, it will be a potential method in regenerative treatment if MSCs derived exosomes are used because MSC can be obtained from Wharton's Jelly which is a waste. Zhang *et al.* [32] proved that exosomes originating from MSCs can help repair cartilage. Exosomes can also help periodontal regeneration by increasing the migration and proliferation of the periodontal ligament cells [33]. However, the use of exosomes as a therapeutic agent currently still has obstacles, including the small number of exosomes produced [31,32,34].

Compared with stem cells or cell-based applications, the use of exosomes provides key advantages over cell-based therapy. The use of exosomes resolves several problems associated with the transplantation of living and proliferative cells population, which cannot be fully controllable *in vivo*. Besides, the immune compatibility, tumorigenicity, emboli formation and the transmission of infections can also be prevented. Moreover, the preparation prior to its applications can be evaluated for its safety, dosage and potency in a method analogous to the preparation of conventional pharmaceutical agents and the use of toxic cryo-preservative agent can also be avoided [35,36]. Eiro and co-workers [35] also predicted that mass production is possible through tailor-made cell lines under controlled laboratory conditions, allow it to provide off-the-shelf exosome therapies immediately.

## Biogenesis, functions & potential of exosomes

As previously described, the secretome of MSC consists of soluble factors and EVs. The EV contains biologically active molecules, such as growth factors, cytokines and functional RNAs. Exosomes are released by MSC. At the time of this release, biological factors contained in the exosomes can exert an effect on the cells of the cellular environment or reach distant organs via the bloodstream, including the central nervous system, which processes depend on the permeability of the blood–brain barrier.

Exosomes are originated from endosomal and the size ranges from 30 to 100 nm [6,28], or 50 to 100 nm according to the Minimal Information for Studies of Extracellular Vesicles (MISEV) [26]. Its biogenesis is known to be regulated by specific cellular pathways [37]. Constitutive exocytosis, which occurs in almost all cell types and involved in the secretion of newly synthesized protein, triggers exosomes release. Exocytosis is initiated by invagination of endocytic vesicles that fused with early endosome, continued by endosomal formation [38]. In this way, exosomes are the result of the secretion process of the endosomal components of the cells.

**Figure 4.**
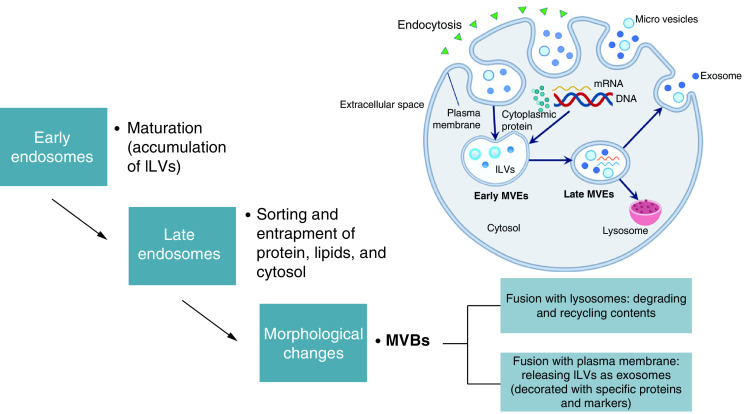
Biogenesis of extracellular vesicles. Microvesicles and apoptotic bodies are originated from plasma membrane, while exosomes are derived from the endosomal compartments. Through an ESCRT-dependent pathway, proteins, lipids, nucleic acids and other cargo are sequestered within the ILVs. The MVBs which fuse with plasma membrane will release ILVs into extracellular space as exosomes. ESCRT: Endosomal sorting complex required for transport; ILV: Intraluminal vesicle; MVB: Multivesicular body.

Unlike the microvesicles or ectosomes and apoptotic bodies which are directly shed from the plasma membrane, exosomes are released upon the fusion of late endosomes and multivesicular bodies with the plasma membrane [39]. A highly dynamic endocytic pathway started the first step of exosomes release. The release is started by the accumulation of intraluminal vesicles (ILVs) as early endosomes mature into late endosomes. The ILVs sort and entrap proteins, lipids and cytosol within the late endosomes. The endosomal sorting complex required for transport (ESCRT) is found to control these initial steps of exosomes secretion [40]. The entrapment leads to morphological changes that result in multivesicular bodies (MVBs), as described in the previous studies [41–43]. In most cases, MVBs fuse with lysosome for the degrading and recycling their contents. Certain MVBs are patterned with specific proteins and markers to ensure the fusion with the plasma membrane, allow the release of their contents to extracellular space, and become known as exosomes. This mechanism is dependent, controlled by ESCRT and involves 30 different proteins which help sequester specific biomolecules in the MVBs and guide their releases through the plasma membrane as exosomes [39,43–45]. The diagrammatic cascade of exosomes biogenesis is shown in Figure 4.

Exosomes associate with the progression of diseases, among them are neurodegenerative disease, cardiovascular diseases and cancer. The association is related to the involvement of exosomes in many physiological processes such as antigen presentation, RNA transfer or tissue repair [31–35,46]. Evidence from the previous studies shows that exosomes have specialized functions and key roles in coagulation, intercellular signaling and waste products management. Their functions include immune regulation, vascular regeneration promoting, mediation of cell proliferation, differentiation, migration, and apoptosis, preserving the body physiological condition and partaking in disease processes [20].

MSC-derived exosomes (MSC-exosomes) exhibit high potential for cell-free-based therapy in regenerative medicine. Since MSCs derived exosomes have the characteristics of the resource cells, thus it can promote cell self-repair, regenerate tissue, restore homeostasis of the tissue and accelerate wound healing [47]. A lot of research shows that MSCs have a strong ability to produce exosomes [45–48] and MSCs derived exosomes are believed as the main effective paracrine component which plays almost equivalent biological effects to those of whole MSCs.

In comparison with the whole MSCs, exosomes fuse directly with the targeted cells, thus exhibit more intense biological effects. Exosomes derived from MSCs can be easily stored and transported at -70°C for a long time. Their main components are effectively protected by the exosome's plasma membrane which make them not easily destroyed. The concentration, dosage, route, and time of use are also easily controlled. Moreover, there is no risk of immune rejection and tumorigenesis caused by cell transplantation therapy [47].

## Potential application of exosomes in regenerative dentistry

An engine search by MEDLINE (PubMed) database with relevant key words such as exosome, regenerative therapy, regeneration, tissue engineering and/or dentistry was used to search relevant articles to this review published up to 31 January 2021. When the terms ‘exosome’ AND ‘dentistry’ AND ‘regeneration’ were used, 60 articles were found. After the screening of the titles, among 60 there were 27 titles directly related or relevant to the study. Among 27, one article provided an experiment related to antibacterial activity of exosome, but after further reading, it was found that the authors only used exosome-liked vesicles from three bee products, in other words, honey, royal jelly and bee pollen [49]. A review article on the exosome derived from saliva [50] was also excluded from the study. Although the article [50] provided description on regeneration of wound healing by salivary exosome, but it was considered not directly relevant to this review. There were also other four review articles concerning exosome or cell-free-based tissue engineering, regenerative therapy and nanotechnology [51–54] which are not specifically related to regenerative dentistry. Therefore, we finally excluded them from the study, remaining 21 articles for further analysis. Table 2 summarizes search results from the 21 articles [55–75].

**Table 2.** Summary of the search results from the MEDLINE (PubMed) database for the articles concerning the application of exosome in regenerative dentistry.

Functions in dentistry	Summary	Type of the study	Study	Ref.
To regenerate periodontal ligament (PDL)	MSC exosome-loaded collagen-sponge-enhanced periodontal regeneration in an immunocompetent rat periodontal defect model	*In vivo*	Chew *et al.*	[55]
To induce bone, cartilage, dentin, mucosa and pulp tissue formation	Functions of MSC exosome in relation to oral and craniofacial tissue engineering	Review	Cooper *et al.*	[56]
To repair critical size osteochondral defects	Exosome enhanced matrix synthesis and a regenerative immune phenotype in osteochondral defect	*In vivo*	Zhang *et al.*	[57]
	Exosome derived human embryonic MSCs promoted osteochondral regeneration	*In vivo*	Zhang *et al.*	[58]
To enhance angiogenesis in oral wounds	Possible implication of exosome for therapeutic induction of angiogenesis in the oral wounds	Review	Zimta *et al.*	[59]
To function as small molecule drug to enhance chondrogenesis	Exosome improved efficient delivery of kartogenin to synovial fluid derived MSCs for chondrogenic differentiation	*In vitro* and *in vivo*	Xu *et al.*	[60]
To treat OA (osteoarthritis) in TMJ (temporo mandibular joint)	Potential of exosome in regenerating cartilage and osseous compartments in TMJ, thus restoring injured, dysfunction, and pain tissues	Review	Lee *et al.*	[61]
	Exosome attenuated inflammation and restored matrix homeostasis	*In vitro*	Zhang *et al.*	[62]
To control dental-pulp derived pain and inflammation	Potential of exosome to modulate thermo-sensitive receptor potential cation channel in pain and inflammation management in everyday dental practices	Review	Schuh *et al.*	[63]
To promote oral mucosal wound healing	Exosome isolated from clinical grade production of oral mucosal epithelial cells stimulated epithelial regeneration and showed pro-regenerative effects on skin wound healing	*In vitro* and *in vivo*	Sjöqvist *et al.*	[64]
To enhance endodontics and pulp regeneration	Potential of exosome as an approach to enhance regenerative endodontics	Review	Tatullo *et al.*	[65]
	Potential of exosome to trigger pulp regeneration (including pulp angiogenesis), regulate proliferation, migration and differentiation and provide neuroprotection	Review	Yu *et al.*	[66]
	Exosome was isolated from DPCs culture supernatant and examined on its roles to HUVEC proliferation, pro-angiogenic factors expression and tubular formation. It was found that exosome-derived DPCs have vital roles in angiogenesis and tubular morphogenesis	*In vitro*	Xian *et al.*	[67]
To enhance cutaneous wound healing	Exosome from neonatal serum used to pre-treat MSCs improved MSCs biological functions in enhancing angiogenesis	*In vitro*	Qiu *et al.*	[68]
To enhance nerve regeneration, increase number and diameter of nerve fibers and promote myelin formation	Exosome was isolated from gingival MSCs, combined with biodegradable chitin conduits and applied to rat sciatic nerve defect. Number and diameter of nerve fibers increased significantly. There was also significant increased proliferation of Schwann cells and dorsal root ganglions by the treatment	*In vivo*	Rao *et al.*	[69]
To inhibit cancer growth	Potential of exosome to inhibit cancer growth since it may transduce apoptosis-inducing factors	Review	Stefanska *et al.*	[70]
To regulate bone remodeling and function as therapeutics agent in orthodontics	Potential of exosome to enhance communication networks integrating bone cells osteoblast, osteoclast, osteocyte) and linking bone to other tissues. These potentials are significant to augment bone remodeling associated with orthodontic force application or required for the repair of craniofacial bone	Review	Holliday *et al.*	[71]
To induce dentinogenesis in regenerative endodontics procedure	Stem cells from apical papilla derived exosomes were applied into the root fragment containing bone marrow MSCs and transplanted subcutaneously into immunodeficient mice. It was observed that dental-pulp like tissues were present and newly formed dentine was deposited onto the existing root canal. It was also observed that dentine sialophosphoprotein and mineralized nodule were significantly increased	*In vitro* and *in vivo*	Zhuang *et al.*	[72]
To regenerate and repair tissue through cell-free-based tissue engineering with sustained release capability	Potential of exosome as mediators for tissue regeneration. The review describes exosome involvement in a multitude of physiological processes, such as development, cell differentiation and angiogenesis	Review	Alqurashi *et al.*	[73]
To accelerate craniofacial regeneration when combined with three-dimensional block co-polymer	Exosome derived from human dental pulp stem cells loaded into biodegradable triblock copolymer microspheres of poly(lactic-co-glycolic-acid) or PLGA and poly(ethylene glycol) or PEG facilitated bone marrow stromal cells. It was also observed that direct insertion of the construct into calvaria defect of the mouse accelerated bone healing *in vivo*	*In vitro* and *in vivo*	Swanson *et al.*	[74]
To regenerate salivary gland	Potential of exosome to ameliorate salivary gland injury by combination of three-dimensional bioprinting or bio assembly spheroid or organoid cell transplantation	Review	Chansaenroj *et al.*	[75]

DPC: Dental pulp cell; MSC: Mesenchymal stem cell.

In his review, Cooper and co-workers [56] mentioned that exosomes carry with them informative cargo from the MSCs to targeted cells. The informative cargo is needed to regulate fundamental cellular processes for lineage-specific differentiation, migration and apoptosis. Regarding bone regeneration, key protein factors carried by exosomes will mediate a series of signaling pathway [76]. For example, an important transcription factor RUNX2 in exosome will promote differentiation of pluripotent stem cells into osteoblast and at the same time inhibit osteoblast maturation [77] or repress osteogenic transcription factors such as OPN, BSP, OSX, dan OCN [78]. Table 3 shows key bone regeneration factors mediated by protein carried by exosomes [76–106].

**Table 3.** Key bone regeneration factors mediated by protein or cytokine carried by exosomes.

Bone regeneration factor	Function	Study	Ref.
RUNX2	Key transcription factor for the differentiation of stem cells into osteoblast and inhibition of osteoblast maturation by suppression of OPN, BSP, OSX and OCN expression	Wang *et al.*; Deng *et al.*	[77,78]
PI3K-AKT	Key transcription factor with phosphatidylinositol 3-kinase (PI3K) and Akt/protein kinase B proteins involved. This signal transduction pathway promotes metabolism, proliferation, cell survival, growth and angiogenesis in response to extracellular signals	Xu *et al.*; Zhao *et al.*	[79,80]
Wnt	Key transcription factor for signaling pathway related to bone remodeling and repair. Wnt signaling system is also known to be a key factor to maintain bone mass	Komiya and Habbas; Issack *et al.*; Grigorie *et al.*; De Santis *et al.*	[81–84]
RANKL-RANK	Key signaling pathway responsible for homeostasis of bone metabolism determined by dynamic balance between osteoblast and osteoclast	Theoleyre *et al.*; Wada *et al.*; Leibbrandt *et al.*; Huynh *et al.*	[85–88]
BMP2	It is multi-functional growth factors which belong to superfamily TGF-β. The BMPs play critical roles in cartilage development, and specifically has been utilized for the therapeutics of bone defects, bone fractures, osteoporosis, spinal fusion and root canal surgery	Chen *et al.*	[89]
BMP9	BMP9 is known to have highest osteogenic potentials compared with other BMPs family. However, it is also revealed that BMP9 exerts broad range biological functions such as adipogenesis, angiogenesis, neurogenesis, oncogenesis and/or tumorigenesis and metabolism	Mostafa *et al.*; Bharadwaz and Jayasuriya	[90,91]
SPP1 (OPN)	SPP1 is also known as BSP1 or OPN. Among its diverse biological functions, OPN is known to regulate biomineralization because its calcium binding sites. As a member of SIBLING (Small Integrin-Binding Ligand, N-linked Glycoprotein) family, it can interact directly with extracellular matrix including fibronectin	Chen *et al.*; Mukherjee *et al.*; Fisher *et al.*; White *et al.*; Lund *et al.*; Singh *et al.*; Si *et al.*	[92–98]
OCN	Produced by osteoblast, OCN is the most abundant non-collagenous protein in bone. It regulates bone mineralization and coordinates mineral ions homeostasis. Bone quality is regulated by OCN because it aligns biological apatite parallel to the collagen fibrils	Wei and Karsenty; Komori	[99,100]
COL1	Type I collagen is the most abundant collagen and a key structural composition of bone tissue that is also expressed in almost all connective tissue as predominant component of interstitial tissue membrane	Henriksen and Karsdal	[101]
TGFβ1	TGFβ1 is abundant in bone, responsible for bone formation and resorption. It stimulates matrix protein synthesis and at the same time inhibits both osteoclast formation and activity	Bonewald and Mundy; Mundy	[102,103]
VEGF	This growth factor, VEGF belongs to PDGF super family. It regulates angiogenesis and vascular permeability	Risau; Shibuya	[104,105]
PDGF	When being activated, PDGF stimulates cell growth, changes cell shape by reorganization of actin filament and affects chemotaxis which directs cells motility. Its role is important during embryonic development and wound healing	Heldin and Westermark	[106]

The table is summarized from a chapter written by Yang *et al.* [76].

BMP: Bone morphogenetic protein; BSP: Bone sialoprotein; COL1: Type I collagen; OCN: Osteocalcin; OPN: Osteopontin; OSX: Osterix; SPP1: Secreted phosphoprotein 1

**Figure 5.**
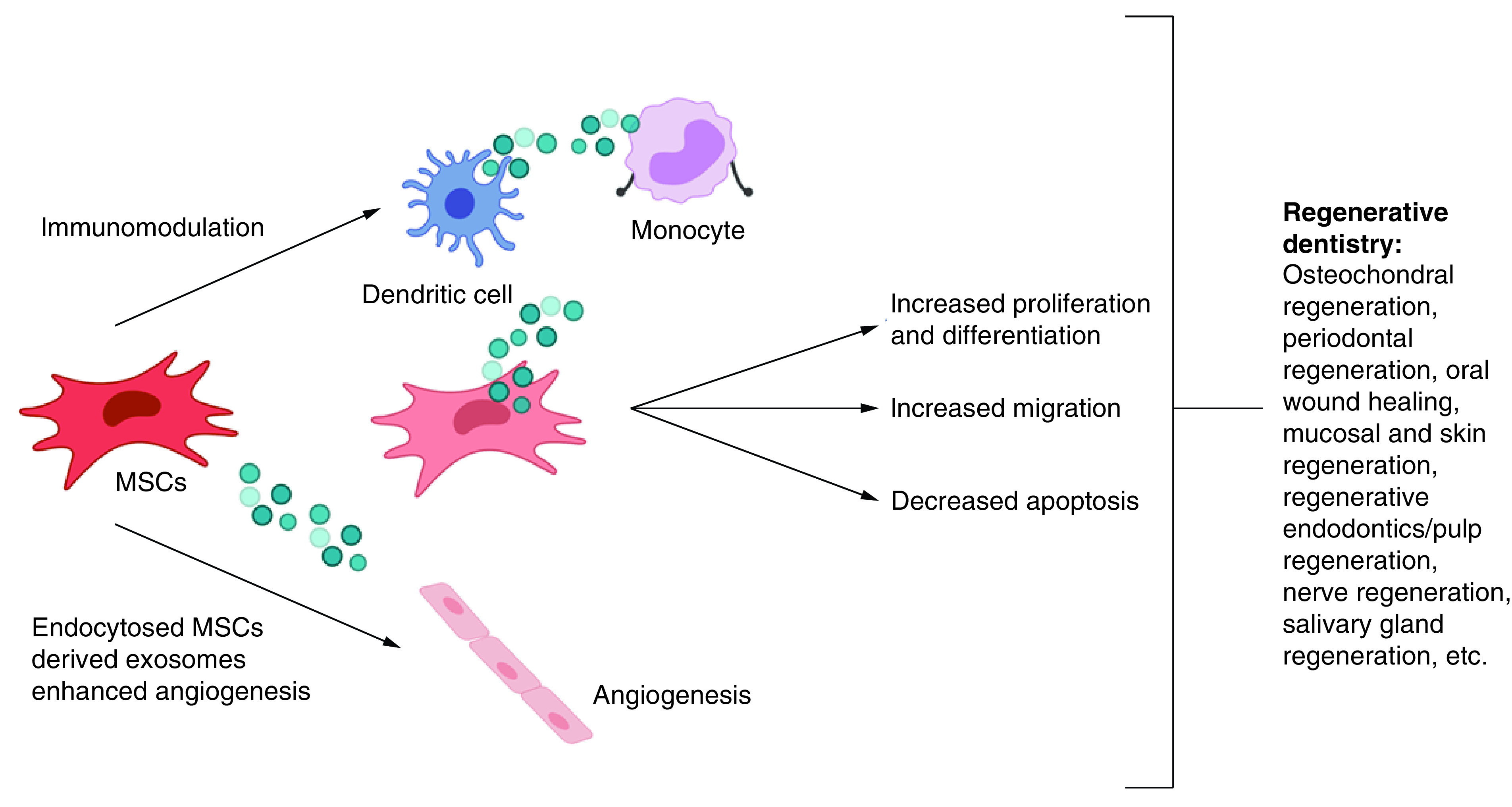
Mesenchymal stem cells derived exosomes with the potential to enhance regeneration in clinical dentistry.

It is also notified that generally exosome enhanced regeneration by increasing cellular mobilization and proliferation. In case of PDL, Chew and co-workers used PDL cell cultures and found that exosome increased PDL migration and proliferation through CD73-mediated adenosine receptor activation of pro-survival AKT and ERK signaling [55]. Meanwhile, the mechanism of exosomes involvement in promoting bone regeneration process can be observed from the process in which proteins carried by exosomes upregulate bone regeneration factors, as depicted in Figure 5. For example, exosome may induce high expression of BMP2 which in turn promotes osteogenic differentiation and osteogenesis by cascade activation of OSX factor [107]. Similarly, when OPN or Type I collagen is upregulated by exosome, bone mineralization cascade will be activated followed by other bone regeneration processes. High expression of other bone regeneration factors such as BMP9, TGF-b1, VEGF and PDGF induced by exosomes will activate osteogenic differentiation and angiogenesis pathway to enhance bone regeneration [76]. In view of the potential of exosome in PDL and bone regeneration, the implication in regenerative dentistry can be clearly elaborated, such as for craniofacial regeneration, implant dentistry, oral-maxillofacial regeneration in general, PDL regeneration, and in orthodontics for application related to bone remodeling control to avoid relapse post orthodontics treatment.

Related to the role of exosome in cartilage regeneration, firstly it is known that cartilage is a connective tissue with limited capacity for intrinsic regeneration upon injury or lesions. Cartilage injury can also be further aggravated by several joint diseases such as osteoarthritis (OA) and rheumatoid arthritis (RA). In clinics, OA is characterized by joint pain, tenderness, crepitus, stiffness, limitation of movement due to occasional effusion with various degrees of local inflammation [108,109]. It is the most frequent chronic joint disease, with progressive breakdown of articular cartilage [110]. Meanwhile, RA is characterized by dysregulated inflammatory processes in the synovium of the joint. The inflammatory process eventually leads to the destruction of cartilaginous and bony elements of the joint, resulting pain and disability [111]. It is an autoimmune disorder in which dysregulated inflammation and T-cells induce pain and joint degradation [112].

Zhang *et al.* [58] demonstrated that weekly intra-articular injections of human embryonic MSC exosome has successfully induced an orderly cartilage regeneration and subchondral bone in a rat model, marked by the development of hyaline cartilage and underlying subchondral bone. The osteochondral defect repair is characterized by increased cellular proliferation and infiltration, enhanced matrix synthesis, and a regenerative immune phenotype [57,58]. The results of the study by Zhang and co-workers [58] corroborated with a previous study used exosome-derived synovial fibroblasts of RA patient which found that exosome from RA synovial fibroblast of patient (RASF) has a membrane bound form of TNF-a, which leads to apoptotic resistance of T-cells in RA because the lack of apoptotic machinery for T-cells progresses RA [113]. When apoptotic resistance of T-cells increases, there would be delayed onset of RA. In regenerative dentistry, these findings are relevant for the treatment of TMJ disorders because TMJ injury, dysfunction, and pain are closely related to the manifestation of OA and RA in dentistry [61,62].

Generally, during regeneration process, vascularization is essential because new blood vessel formation improves diffusion of oxygen and nutrients in the regeneration area. It has been demonstrated that exosome can stimulate proliferation, migration, and tube formation of endothelial cells (ECs) [114,115]. As it is widely known, angiogenesis is a process of blood vessel formation and stability which is controlled and dictated by VEGF [59,104,105]. Beside having action on vascular endothelial cells, VEGF also enhances bone development by stimulating vascular endothelial cells. Therefore, in some studies, it was known that osteogenesis is closely related to vascularization through cell-to-cell communication between vascular endothelial cells and osteoblasts. In other words, sufficient vascularization is considerably promoting osteogenesis [116–119]. Moreover, it is also revealed from the previous study that exosome-derived MSCs contained abundant levels of VEGF, which enhanced angiogenesis and contributed to periodontal tissue [73,120], oral epithelial [64], and dental pulp or endodontics [65] regenerations.

**Figure 6.**
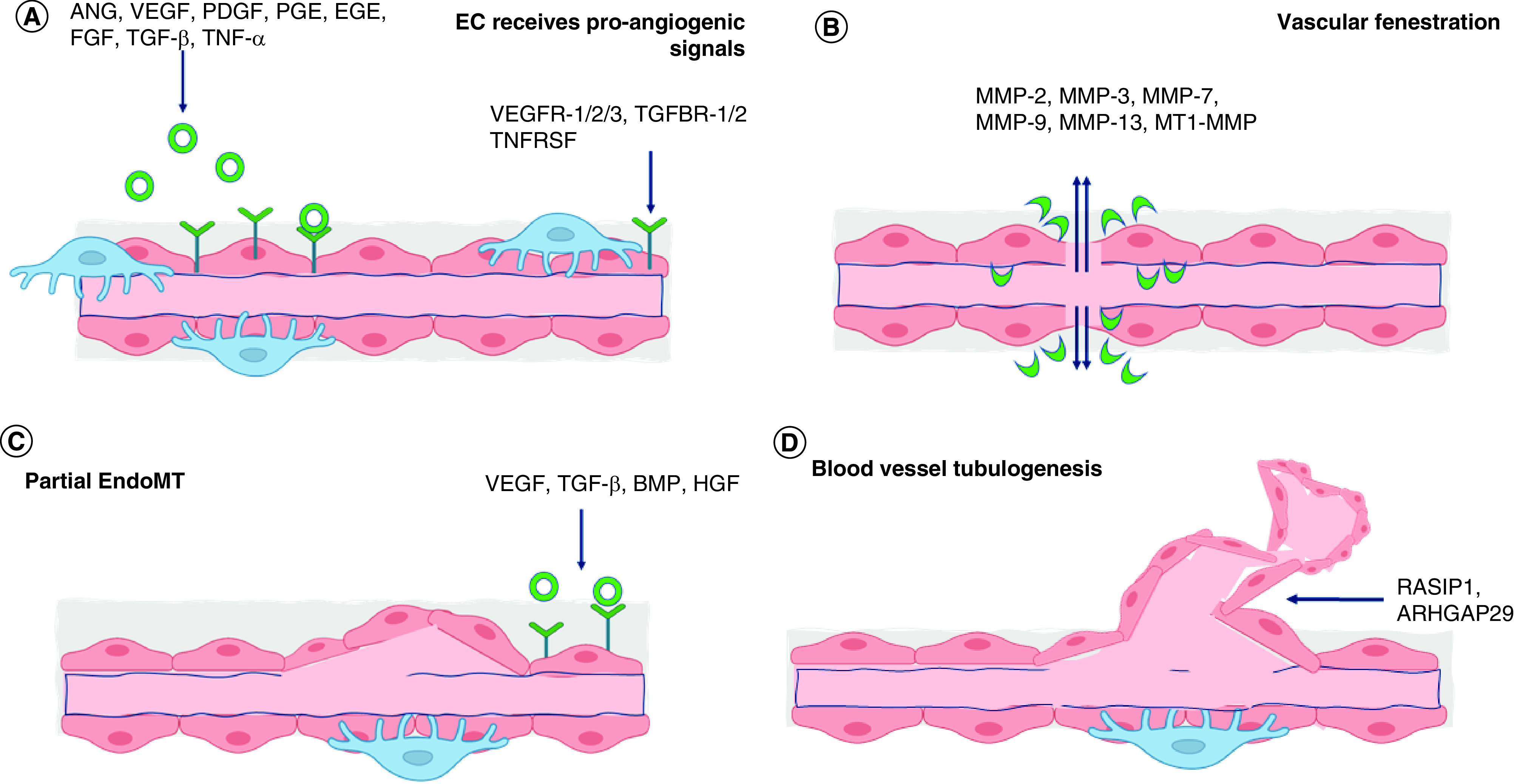
Angiogenesis is composed of several stages, as depicted schematically. The diagram is modified from and referred to the work Zimta *et al.* [59]. At first stage, pro-angiogenic factors, which bind to their corresponding receptors, are released by surrounding cells into local environment. **(A)** The ECs found at the outer surface of a blood vessel receive pro-angiogenic signals from ANG, VEGF, PDGF, PGF, EGF, FGF, TGF-β, and TTNF-α. On the surface of EC, there are several receptors corresponding to pro-angiogenic factors, for instance VEGFR1/2/3, TGFBR1/2, and TNFRSF. After signal transduction in the EC, cells start to proliferate and produce metalloproteinases (MMP). **(B)** Concurrently, blood vessel pores increase in size and fenestration happens. This will enable MMPs to escape from the blood vessels and degrade basement membrane. **(C)** The ECs start to migrate. This process is called partial endothelial to mesenchymal transition (partial EndoMT), proliferate at the fenestration area, and result a new blood vessel budding. **(D)** As the new tube forms, multiple signals from the environment such as RASIP1 and ARHGAP29 will enhance the development of 3D structure and organization of the newly formed network. By the final stage of the process, pericytes found at the exterior of the blood vessel responsible for blood vessel contraction begin to populate the newly formed network. EC: Endothelial cell.

As depicted schematically in Figure 6, angiogenesis is composed of several stages [59]. Regarding angiogenesis which leads to neovascularization in regeneration area, exosomes are known as mediators for intercellular communication and can be used to maximize the local pro-angiogenic potential at the wound site. Abundant number of pro- and anti-angiogenic microRNAs were found [59] and this modulates the behavior of endothelial cells and could be included in the design of exosome delivery of pro-angiogenic factors. Exosome with its miRNA cargoes direct the process during neovascularization, for example miRNA-21 activates AKT and ERK pathway, thus leading to the VEGF-increased production. Besides, when generating exosomes, MSCs are maintained in hypoxic conditions. This hypoxic condition is predicted to stimulate enhancement of pro-angiogenic capacity of the exosomes [121]. The underlying mechanism is possibly due to overloading of exosomes with miR-135b which targets the factor inhibiting HIF-1 (FIH-1) gene, an inhibitor of hypoxia-inducible factor 1 (HIF-1), known as pro-angiogenic factor [122].

Beside cartilage, bone, oral mucosal, and PDL regenerations, nerve regeneration pays a lot of attention in dentistry due to prevalent cases of nerve injuries caused by oral-maxillofacial traumas and/or surgeries. From several studies, it was observed that exosome could stimulate Schwann cell proliferation and increased expression of cyclin Ki67 as an indication of exosome potentials in enhancing neurite length of dorsal root ganglion (DRG) neurons [69,123]. The capability of exosome in enhancing nerve regeneration is due to the presence of neuronal growth factors such as brain-derived neurotrophic factor (BDNF), insulin growth factor-1 (IGF-1), nerve growth factor (NGF), fibroblast growth factor-1 (FGF-1), and glial cell-derived neurotrophic factor (GDNF) [124,125]. Based on the presence of key neural growth factors, exosome could potentially provide new approaches to nerve regeneration in medicine and dentistry but further research to uncover underlying neurogenesis mechanism and roles of specific signaling molecules in relation to neural regeneration pathways are needed.

## Therapeutic potential of exosome in healing & regeneration process

From the previous description, it can be concluded that exosomes have potential in the process of regeneration, thus exosomes can be used as therapeutics agent in medicine and dentistry. These potentials cannot be separated from the three overlapping stages in regeneration process: inflammation, proliferation, and remodeling phases. Inflammation is a human body self-defense mechanism against harmful stimuli. It is a regulated acute response beneficial for wound healing [126]. If the inflammation phase were chronic and dysregulated, wound healing would be delayed and it would promote fibrosis, excessive scar formation, and inhibited proliferation or re-epithelization [127].

During the inflammation process, macrophages are a major component of the mononuclear phagocyte system and play critical roles in initiation, maintenance, and resolution of inflammation. Macrophage functions as antigen presenting cells and produce cytokine and growth factors for immunomodulation [128]. Macrophages are activated and deactivated during the inflammatory process. Activation of inflammation induces M1 phenotype to release signals, including cytokines such as interferon gamma (IFN-g), tumor necrosing factor (TNF), bacterial lipopolysaccharide (LPS), ECM proteins, and other chemical mediators. In concert with microbial products, such as LPS, and cytokines, such as TNF, IFN-g will activate M1 [129] indicated by high interleukin-12 (IL-12) and IL-23, as well as high toxic intermediates such as nitric oxide (NO) and reactive oxygen intermediates (ROI) productions [130]. Inflammation deactivation happens by removal of mediators to permit host to repair damage tissues in which M2 phenotype releases anti-inflammatory cytokines (interleukin 10 or IL-10 and TGF-b) and cytokines antagonist [128–130].

**Figure 7.**
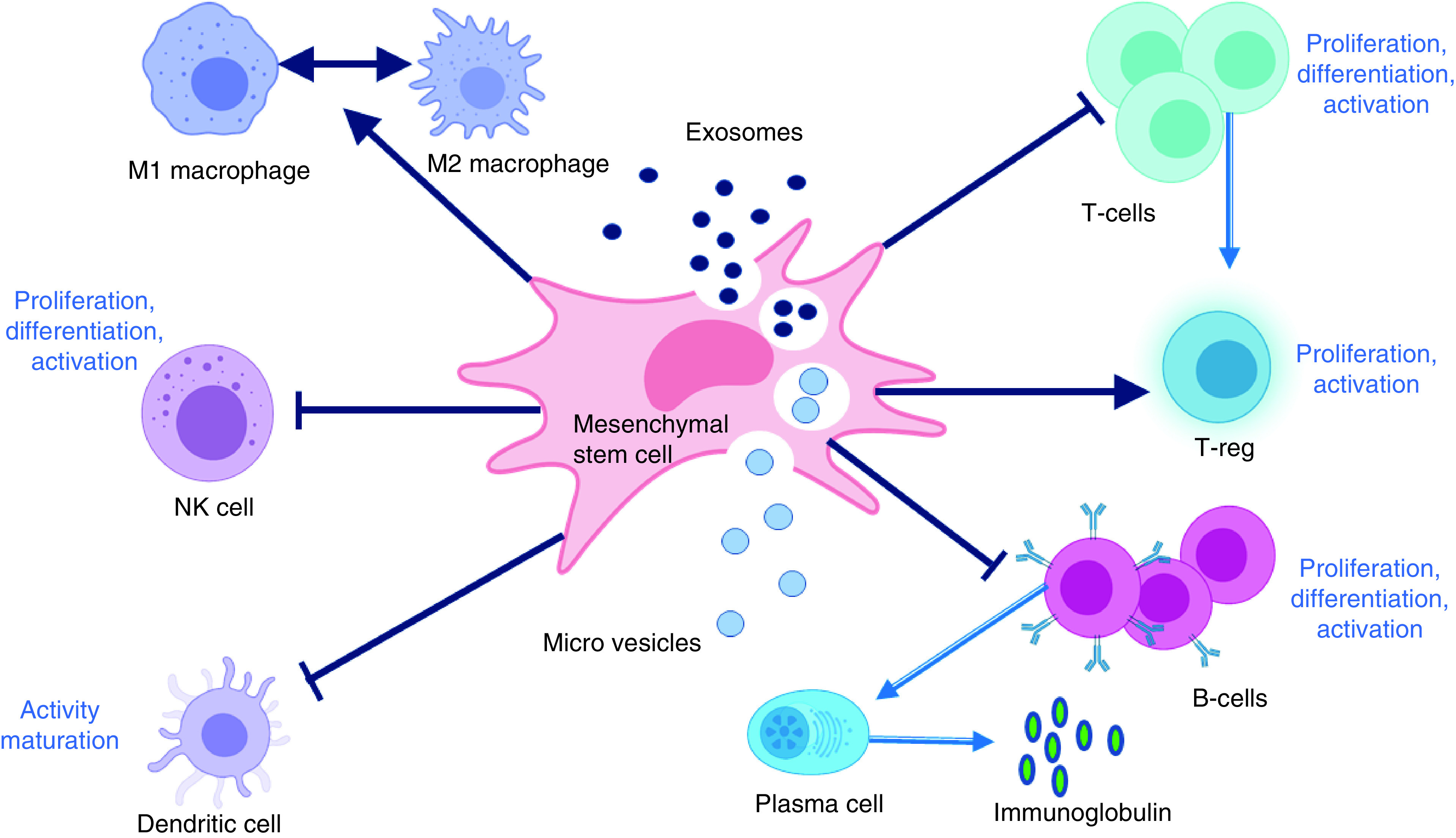
Exosomes and microvesicles (MSC's derived EV) exert immunomodulatory effect on innate and adaptive immune reactions mediated by many immune cells (T and B lymphocytes, natural killer cells, dendritic cells, and macrophages). Exosomes and microvesicles can inhibit the proliferation, differentiation, and activation of T, B, and natural killer cells and the pathogen-presenting function of dendritic cells and macrophages. Macrophage polarization can also be regulated under different microenvironment in accordance with EVs application. Figure was derived and further modified from Wang and co-workers [133].

Since macrophages produce a wide range biologically active molecules to autoregulate in the inflammatory process, therapeutic interventions using exosome which targets macrophage may open new avenues for inflammatory disease control. Exosome can regulate activation, differentiation, and proliferation of B-lymphocytes and suppress T-lymphocyte proliferation at the same time. It can also convert activated T-lymphocytes into T-regulatory phenotype to exert immunosuppressive effects [126,131,132], as depicted in Figure 7, summarized from a review on the role of EVs in autoimmunity [133]. The regulation of inflammatory factors plays important roles in regeneration process. With the capacity to deliver a cargo of protein, lipids, nucleic acids, or other cellular components to neighboring or distant cells, exosomes may polarize the inflammatory response through down-regulation of pro-inflammatory enzymes like inducible NO, cyclooxygenase (COX)-2 and of cytokines such as TNF-a, IL-1b, monocyte chemoattractant protein (MCP)-1. Table 4 summarizes possible mechanism of exosomes in immune regulation and inflammatory responses, as the key points for therapeutics and regenerative treatment [124,129–132].

**Table 4.** Possible mechanism of exosomes in immune regulation and inflammatory responses.

Target	Effect	Mechanism
CD4^+^ and CD8^+^ T-cells	Inhibiting differentiation toward effector or memory T-cell phenotypes	Mediated by anti-CD3/CD2/CD28 stimulation
T-cells in general	Inhibiting activation	Reduction of interferon-gamma (IFN-γ) secretion
Macrophage	Increasing mRNA levels of M2-related arginase-1 and interleukin-10 (IL-10)	
	Inducing macrophage polarization toward anti-inflammatory M2 phenotypes	Transactivation of arginase-1 by active signal transducer and activator of transcription 3 (STAT-3)
	Inhibiting macrophage inflammatory responses	Stimulated by lipopolysaccharide (LPS) and IFN-γ
Inflammatory response (*in vivo* in mouse model)	Regulating inflammatory responses	Reduction of inflammatory cytokines sech as IL-4, IL-23, IL-31, and tumor necrosis factor-alpha (TNF-α)
Inflammatory response (*in vivo* in mice after sepsis syndrome)	Improving survival and suppressing inflammatory responses	Increase inflammatory mediators such as MMP-9, macrophage migration inhibitory factor, TNF-α, nuclear factor kappa-B (NF kappa-B), and IL-1β

References [124,129–132] used as sources of information.

During proliferation phase, angiogenesis plays crucial roles in wound healing and repair. For this, MSCs derived exosomes are enriched with various angiogenesis related proteins and other miRNAs that could activate signaling pathways in endothelial cells, including up regulating angiogenesis related molecules found in vascular endothelial cells such as VEGF, VEGF receptor (VEGFR), FGF-1, E-selectin, angiopoetin-1 and endothelial nitric oxide synthase (eNOS), chemokine ligand 16 and IL-8 [126]. Furthermore, ECM reconstruction is the key point for tissue reconstruction during remodeling phase. It was observed that exosomes promoted synthesis of type I collagen, type III collagen and elastin during the early stage and inhibited collagen synthesis in the late stage of remodeling to inhibit scar tissue formation [126]. In this context MSCs derived exosomes will be potential candidate as a therapeutic agent to regulate inflammatory, proliferation and remodeling phase in tissue regeneration.

Moreover, because of their ideal native structure and characteristics, exosome is also indicated as a promising nanocarriers for clinical use due to its ideal small size to penetrate deep tissues, slightly negative zeta potential for long circulation, deformable cytoskeleton and similarity to cell membranes [133,134]. Exosomes can also exhibit an increased capacity to escape degradation or clearance by immune system [133].

## Challenges & strategies

Based on the previous review, the MSCs derived exosomes and their potential as cell-free-based therapy in tissue engineering applied in dentistry, the challenges for the application include extensive research on the identification of the molecules involve in paracrine action of stem cells to open new therapeutics options using exosomes, the production, processing and manufacturing aspects, as well as strategies to develop the cost-effective system for clinical applications.

The initial challenges to be considered are exosome isolation, purification and characterization. In general, exosomes can be isolated from the conditioned media of cultured cells and almost any biological fluids [45]. Comprehensively, Li and co-workers wrote an update on various exosome isolation techniques and described the advantages and disadvantages of isolation techniques based on ultracentrifugation, size, immunoaffinity captured, precipitation and microfluidics [135]. To prepare clinical grade exosome, good manufacturing practices (GMPs) and quality control become an utmost important factor. In view of this, cell source and state including microenvironmental conditions must be kept uniform to provide consistent exosome quality and yield [45].

The development and translational framework of human EV-based therapeutics in general, or specifically exosomes, is regulated under biological medicinal products category [136]. A biological medicine contains one or more active substances made by or derived from biological cells, and to some extent equivalent to biologic drugs, biologicals or biopharmaceuticals [137–140]. Although regulatory framework for manufacturing and clinical trials exists in Europe, Australia and USA, but research related to establishment of special guidelines targeting EV-based therapeutics relevant to isolation, purification, characterization and their valorization is considered important area to be investigated. In the context of exosome-based therapeutics valorization and clinical translation, mechanism of action (MoA) is essential and iterative because the dissection between exosome as an active substance and excipients are important to control the quality and safety of the exosome-based therapy. Further challenges related to the changes and differentiation of residing stem cells surrounding treatment area also need special attention since exosome may influence cell behavior [141,142] including the risk of factors transduction and other cell to cell communication [143]. For clinical application of exosomes in regenerative therapy, currently there has no standardized procedure yet. The isolation and storage are critical, even more the manufacturing requires adequate and appropriate technology with quality system for the safety of both donor and recipients.

In case of manufacturing for instance, although extensive research has been conducted, but still, the practical use of exosome is restricted by limited exosome secretion capability of cells [144]. Moreover, a large dose of exosome is required in actual clinical administration [136–140,144–146]. On the other hands, several studies show that increased intracellular calcium can lead to the formation and/or production of EVs [135]. In view of this, scaffold which contains calcium may resolve the problems by creating microenvironment with a capability to adjust cell exosome production. As shown in the previous study by Wu and co-authors [144], bioactive glass (BG) ceramic ion products significantly promoted MSCs to secrete exosome without changing the morphology, size distribution and internalization of the MSCs, and simultaneously improved their biological functions.

Typical BG is composed of SiO_2_, Na_2_O, CaO and P_2_O_5_ [147,148] which has been widely used for wound repair and regeneration [149–151]. As reported by Wu *et al.* [144], BG ion products significantly enhanced pathways that generate intracellular exosome vesicles and release mature exosome. Wu and co-authors [144] proposed the underlying mechanism that BG ion products enhanced MSCs to generate exosomes by upregulating the expression of nSMase2 and promoted MSCs to release exosomes by upregulating the expression of Rab27a. Simultaneously, the pathways will promote vascularization of the recipient cells by regulating the levels of miR-342-5p and miR-1290 in cargoes.

From the reports [34], it is known that ceramics-based scaffold can affect the behavior of single type of cells and cell–cell interactions to enhance exosome generation. Regarding this aspect, previous research have been extensively conducted to investigate synergetic effects of ceramics-based scaffold and exosome in regenerative treatment [3,17], however, they elaborated more on the therapeutics effects, not on the effect and mechanism on exosome generation by calcium containing construct such as ceramics-based scaffold.

**Figure 8.**
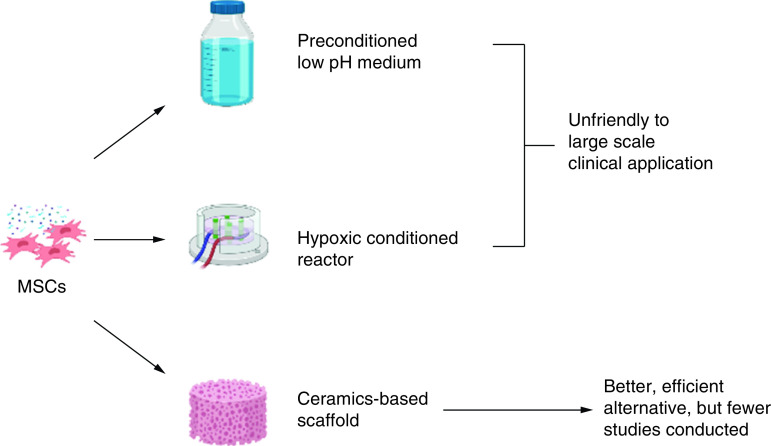
Although limited studies have been conducted, but ceramics-based scaffold has high potential to enhance exosome generation, production and secretion from MSCs due to the release of ion products from the construct such as calcium ions since the increased intracellular calcium can lead to the formation and/or production of extracellular vesicles. The use of ceramics-based scaffold is also beneficial for exosome retention and controlled release delivery system.

**Figure 9.**
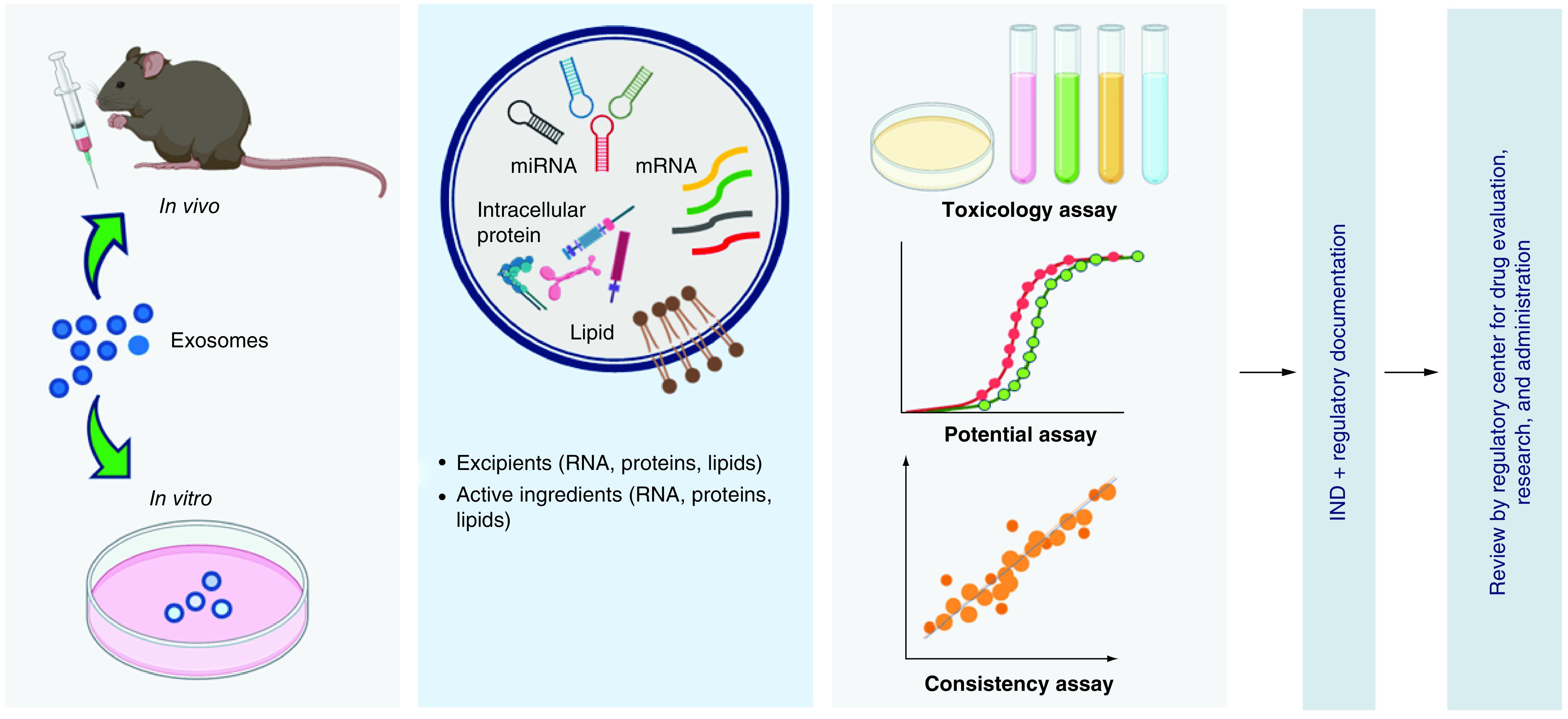
A lot of further *in vitro* and *in vivo* investigations are awaiting regarding isolation, characterization, production and storage of mesenchymal stem cells derived exosome for real clinical application. Mechanism of action, biosafety and biocompatibility on the use of exosomes for cell-free-based tissue engineering and new therapeutics approach are wide open area to be studied before translating exosomes as an investigational new drugs into clinical application.

In view of this, several ceramics-based scaffold can be explored and developed as an effective strategy to either enhance exosome production or yield and improve biological activity of the secreted exosomes. The use of ceramics-based scaffold can be a better alternative strategy to overcome limited exosome secretory from the cells when compared with modulation of cell culture conditions under hypoxia and low pH microenvironment, because it will be difficult to maintain healthy cells in such unfriendly conditions for large-scale clinical applications, as concluded diagrammatically in Figure 8. Therefore, a lot of ceramics-based scaffolds are awaiting to be investigated further as an alternative strategy to enhance exosome production from MSCs or other cells, such as hydroxyapatite, carbonate apatite, calcium carbonate, some calcium orthophosphate including biphasic calcium phosphate, tricalcium phosphate, bioactive glass [3,17] or other mineral-doped scaffold [152]. Specifically, ceramics-based scaffold can function as biomaterial for exosome retention and delivery vehicles for exosome to reach targeted cells. Meanwhile, in general challenges in cell-free-based tissue engineering therapy and therapeutics using exosomes in dentistry and medicine are summarized in Figure 9.

## Conclusion

Clinical application on the use of MSC is limited due to its inherent heterogenicity, variation associated with cell expansion and risk of unwanted differentiation. Therefore, nowadays cell-free-based therapy with MSCs derived exosomes is considered an alternative treatment in dental tissue engineering. This is because exosomes carry with them informative cargos from the MSCs to targeted cells, which is needed to regulate fundamental cellular processes for lineage-specific proliferation, differentiation, migration, apoptosis and modulation of a series of signaling pathway. In the current state, exosome is found to be potential candidate as a therapeutic agent to regulate inflammatory, proliferation and remodeling phase in tissue regeneration. As a conclusion, exosomes could potentially provide new approaches to dental tissue engineering, but further research to uncover underlying mechanism and roles of specific signaling molecules in relation to dental tissue regeneration pathways are needed.

In addition to the first conclusion, currently there has no standardized procedure for the isolation, storage, and manufacturing technology with quality system for the safety of both donor and recipients in large-scale valorization. In case of manufacturing for instance, although extensive research has been conducted, but still, the practical use of exosome is restricted by limited exosome secretion capability of cells. The limitation on the application of exosome in clinics can be resolved by ceramics-based scaffold which can affect the behavior of single type of cells and cell–cell interactions to enhance exosome generation. Therefore, a lot of further investigations are awaiting to develop ceramics-based scaffold that functions in large-scale clinical application for cell-free-based therapy with exosomes to provide better alternative strategy to overcome limited exosome secretory from the cells, because ceramics-based scaffold produces important ions to enhance production and secretion of exosomes from the cells.

## Future perspective

Exosomes are potential as an alternative future treatment in dental tissue engineering and in regenerative medicine. Exosomes carry with them informative cargos from the MSCs to regulate fundamental cellular processes for lineage-specific proliferation, differentiation, migration, apoptosis and modulation of a series of signaling pathway in the targeted cells. Because of that, exosomes are very potential for next generation therapeutic agent to regulate inflammatory, proliferation and remodeling phase in tissue regeneration. However, to date, there are still limited studies conducted to uncover underlying mechanism and roles of specific signaling molecules in relation to dental tissue regeneration pathways. There are also limited research involving exosomes for modulating extracellular signaling and intracellular reprogramming, which have been a significant approaches in tissue engineering.

On the other hands, advances in materials synthesis, protein engineering, molecular self-assembly, bio, micro and nanofabrication technology, nanotechnology as well as micro and nanopatterning technology have contributed to the high possibility of developing future therapy. When it is combined with exosomes technology, this could be resolution in regenerative therapy. Furthermore, progressive advancement in materials sciences may resolve problems and overcome limited capacity of the cells regarding production and secretion of EVs, including exosomes, because hybrid materials containing certain ions can also be designed and directed to enhance generation and secretion of exosomes for large-scale clinical applications.

To achieve future goals, better relevant and predictive *in vitro* or *ex vivo* models are needed to predict efficacy and safety of the cell-free-based therapy with exosomes. In this context, the development of microfluidic organ system known as ‘organ-on-chip’ may be crucial to capture phenomenon happens in body tissues and model diseases to provide accurate personalized medicine, involving a complex of exosomes and other biomolecules with hybrid biomaterials. A microfluidic system will allow researchers to study living tissues and organ in a more complex way and will contribute a lot to uncover underlying concept of exosomes as next generation therapeutic agent, including their novel delivery system. Interdisciplinary research to find out proper hybrid biomaterials to deliver, target, increase production, and increase secretion of exosomes involving bio-nanofabrication are significant to be conducted. Besides, standardized procedure for the isolation, storage, and manufacturing technology with quality system for the safety of both donor and recipients in large-scale valorization must be investigated. Their translational steps into dental clinics and, to large extent, into biomedical applications, are also important to be studied in the near future.

**Executive summary**

- In tissue engineering, the use of exosomes released have become a particular interest for cell-free regenerative therapy due to their epigenetic capacity and cargos.
- To valorize the use of exosomes in large-scale clinical setting, better relevant and predictive *in vitro* or *ex vivo* models are needed to predict efficacy and safety of the cell-free-based therapy with exosomes, as well as to capture phenomenon happens in body tissues and model diseases.
- Interdisciplinary research to find out proper hybrid biomaterials to deliver, target, increase production and increase secretion of exosomes involving bio-nanofabrication are significant to be conducted to uncover underlying concept of exosomes as next generation therapeutic agent.
- Their translational steps into dental clinics and biomedical applications are also important to be studied in the future.
